# Targeting the sAC-Dependent cAMP Pool to Prevent SARS-Cov-2 Infection

**DOI:** 10.3390/cells9091962

**Published:** 2020-08-25

**Authors:** Muhammad Aslam, Yury Ladilov

**Affiliations:** 1 Experimental Cardiology, Department of Internal Medicine, Justus Liebig University, 35392 Giessen, Germany; muhammad.aslam@physiologie.med.uni-giessen.de; 2 DZHK (German Centre for Cardiovascular Research), Department of Cardiology, Kerckhoff Clinic GmbH partner site Rhein-Main, 61231 Bad Nauheim, Germany; 3 Independent Researcher, 42929 Wermelskirchen, Germany

**Keywords:** soluble adenylyl cyclase, cAMP, PKA, EPAC, endocytosis, lysosomes, autophagy, coronavirus

## Abstract

An outbreak of the novel coronavirus (CoV) SARS-CoV-2, the causative agent of COVID-19 respiratory disease, infected millions of people since the end of 2019, led to high-level morbidity and mortality and caused worldwide social and economic disruption. There are currently no antiviral drugs available with proven efficacy or vaccines for its prevention. An understanding of the underlying cellular mechanisms involved in virus replication is essential for repurposing the existing drugs and/or the discovery of new ones. Endocytosis is the important mechanism of entry of CoVs into host cells. Endosomal maturation followed by the fusion with lysosomes are crucial events in endocytosis. Late endosomes and lysosomes are characterized by their acidic pH, which is generated by a proton transporter V-ATPase and required for virus entry via endocytic pathway. The cytoplasmic cAMP pool produced by soluble adenylyl cyclase (sAC) promotes V-ATPase recruitment to endosomes/lysosomes and thus their acidification. In this review, we discuss targeting the sAC-specific cAMP pool as a potential strategy to impair the endocytic entry of the SARS-CoV-2 into the host cell. Furthermore, we consider the potential impact of sAC inhibition on CoV-induced disease via modulation of autophagy and apoptosis.

## 1. SARS-CoV-2-Induced COVID-19

The coronavirus (CoV)-induced disease-2019 (COVID-19) caused by severe acute respiratory syndrome coronavirus 2 (SARS-CoV-2) emerged as an outbreak in late 2019 in Wuhan, China. SARS-CoV-2 is a novel RNA virus sharing homology with previously known SARS and Middle East respiratory syndrome (MERS) viruses. Coronaviruses are enveloped, single-stranded, positive-sense RNA viruses that contain the largest known RNA genome of 27–32 kilobases. Two thirds of the viral genome encode non-structural proteins while the remaining one third encodes four classical viral structural proteins, namely nucleocapsid (N), envelope (E), membrane (M), and spike (S) proteins. The S protein is the most studied viral protein as it contains the receptor-binding domain (RBD) for the ligand on the host cell membrane [1]. The S protein is made up of two subunits, S1 containing the RBD and S2 comprising the stalk. The main receptor for SARS-CoV and SARS-CoV-2 on the surface of the target cells is the angiotensin-converting enzyme 2 (ACE2), a metallopeptidase present on the membrane of several cell types, including alveolar pneumocytes, intestinal enterocytes, kidney proximal tubule cells, vascular endothelial cells, and arterial smooth muscle cells [2,3]. Binding of the viral RBD to ACE2 induces conformational changes in the S protein, leading to cleavage of S1 and S2 by proteases, e.g., TMPRSS2 or cathepsin L, that allows S2 to facilitate viral internalization [4,5,6]. 

The pathology of COVID-19 varies greatly from asymptomatic to fatal conditions such as severe pneumonia or acute respiratory distress syndrome due to a huge spatiotemporal heterogeneity of the organs and multiple mechanisms involved. Entry of SARS-CoV-2 virus into the cell may induce cell injury via two mechanisms. Firstly, the virus causes direct cell injury via induction of apoptosis [7,8,9], pyroptosis [10], and necrosis [11]. Secondly, there are indirect effects, such as suppression of ACE2 expression, resulting in a loss of ACE2 protective effects and dysregulation of the renin-angiotensin-aldosterone system [12], and a “cytokine storm” may be induced by a dysregulated immune response [13,14,15] that leads to endothelial damage [16] and formation of thrombo-emboli [17,18,19]. 

An excessive inflammatory response to SARS-CoV-2 is considered to be the major cause of disease severity and the high fatality rate among COVID-19 patients [13] and is associated with high levels of circulating cytokines, vascular leakage, and immune cell infiltration in the lungs, heart, and kidney [16,20,21,22]. Inflammatory cytokines such as TNF-α, interferon gamma (IFN-γ), interleukin 6 (IL-6), and IL-10 present in the plasma of COVID-19 patients [23,24] induce mitochondrial reactive oxygen species production and impede mitochondrial oxidative phosphorylation and ATP production [25,26] among other effects. This, in turn, may lead to mitochondrial membrane permeabilization, cell death, and multiple organ dysfunction, a syndrome observed in COVID-19 patients [27].

## 2. Role of Endocytosis in SARS-CoV-2 Entry

Among SARS-CoV-2 proteins, the S protein is a key protein in the process of viral entry into the host cell via proteolytic cleavage to form two subunits, S1 and S2 [28,29]. These two subunits have distinct functions: S1 is responsible for receptor binding, whereas S2 is mainly involved in membrane fusion, and both are essential for viral entry into the host cell via the endocytic pathway. Receptor-mediated endocytosis is an advantageous entry route as it can allow the virus (in endosomes) to pass through a barrier of cortical actin and traffic deeply into the cell before expelling its genome to the cytosol. Numerous reports suggest that cathepsin L, a cysteine protease, is essential for the priming of SARS-CoV and SARS-CoV-2 S protein in endosomes for entry into several cell types [29,30,31]. 

It appears that the majority of CoVs, including SARS-CoV-2 in some cell types, enter cells via endocytosis [32]. Interestingly, a recent report by Liu et al. [33] suggested that transport of SARS-CoV-2 from the early endosome to the late endosomes is a requirement to release the viral genome as in the case of SARS-CoV. In agreement, Mingo et al. [34] provided evidence that the entry of Ebola virus and SARS-CoV also display a late entry profile, i.e., in endolysosomes, a hybrid organelle resulting from the fusion of late endosomes and lysosomes. The authors also showed that endolysosomes show higher levels of cathepsin L activity than late endosomes, most likely due to lower pH in endolysosomes. In line with these studies, Ou et al. [29] showed a more than 98% reduction of SARS-CoV-2 S pseudovirion entry in 293/hACE2 cells when the cells were treated with lysosomotropic agents increasing endosomal pH, i.e., ammonium chloride and bafilomycin A. Furthermore, this and another recent study [6] argue for the essential role of cathepsin L in the activation of SARS-CoV-2 S protein, similar to the mechanism reported for SARS-CoV and MERS-CoV [35,36,37]. Altogether, the data indicate that CoVs, including SARS-CoV-2, can enter the target cell through the endocytic pathway in a cathepsin-dependent manner and requires endocytic acidification.

Coronaviruses may also take an endosome-independent route of entry into the host cell. In this case, the viral S protein is cleaved by host cell transmembrane protease serine 2 (TMPRSS2) [35,38]. This exposes the membrane fusion domain of the S protein, allowing the viral envelope to fuse with the host cell plasma membrane and release its RNA directly into the cell cytoplasm [28,39,40]. A novel report by Hoffmann et al. [6] demonstrated an additive effect of cathepsin L and TMPRSS2 inhibitors on the suppression of SARS-CoV-2 entry in epithelial cell line expressing TMPRSS2. However, in primary human cells the role of the TMPRSS2-dependent virus entry pathway is limited by its expression level. Sungnak et al. [41] demonstrated expression of TMPRSS2 only in subset of ACE2-positive human airway epithelial cells. In contrast, cathepsins were more pronouncedly expressed than TMPRSS2, especially cathepsin B, which was expressed in more than 70–90% of ACE2-positive cells. This study further emphasizes the importance of the endocytic pathway in CoV entry.

## 3. Efficacy of Currently Available Lysosomotropic or Endocytosis-Inhibiting Agents to Prevent COVID-19

Due to the key role of endocytosis in the entry of CoVs into the host cell, several attempts have been made to target this process in order to block the entry and/or replication of CoVs. In an in vitro study, lysosomal acidification inhibitor bafilomycin-A1 inhibited entry of porcine delta CoV [42]. In other studies, cathepsin inhibitors Z-FY(t-Bu)-DMK and teicoplanin blocked SARS-CoV and MERS-CoV replication, respectively [37,43]. Likewise, the anti-malarial drug chloroquine inhibits endocytosis via elevating intra-lysosomal pH [44] and thus may inhibit the release of virus from endosomes. Indeed, chloroquine blocked SARS-CoV [45] and SARS-CoV-2 virus replication in Vero E6 cells in vitro [46]. Based on these in vitro data, several drugs clinically approved for other diseases and interfering with host cell endocytosis are being tested for their potential anti-SARS-CoV-2 properties. In this context, chloroquine was the first drug to be tested for its efficacy in COVID-19 patients. In early, small non-randomised clinical trials, chloroquine and its related analog hydroxychloroquine showed efficacy in COVID-19 patients [47], and further large randomised clinical trials were planned worldwide. In a multicentre, open-label, randomized clinical trial (150 patients) conducted in China, administration of high-dose hydroxychloroquine to patients tested positive for SARS-CoV-2 infection with mild to moderate COVID-19 did not result in a significantly higher probability of becoming SARS-CoV-2 negative compared with patients treated with standard of care alone [48]. Moreover, the rate of adverse events in patients treated with hydroxychloroquine was higher. Another multicentre, comparative observational study conducted on 181 hospitalized patients in France found no difference in study outcome between patients treated with hydroxychloroquine and standard of care [49]. Furthermore, 10% of the patients receiving hydroxychloroquine suffered from gross ECG changes, requiring them to stop medication. A retrospective analysis comparing hospitalized COVID-19 patients (*n* = 807) treated with hydroxychloroquine or hydroxychloroquine plus azithromycin versus standard of care in all US veteran health administration medical centers found no difference in reducing the mechanical ventilation. Additionally, the authors observed an increased mortality rate in patients treated with hydroxychloroquine [50]. Recently, a multicentre, open-label, randomized controlled trial was conducted on hospitalized COVID-19 patients in Brazil [51]. The study reported no clinical benefit of hydroxychloroquine alone in combination with azithromycin compared with standard of care in the primary outcome. However, prolongation of the QT interval and elevation of liver enzyme levels was more frequent in hydroxychloroquine- or hydroxychloroquine plus azithromycin-treated patients. Therefore, due to its higher risk of side effects, particularly cardiac toxicity, and its low efficacy, all further clinical trials have been halted [52,53] and its usage for treatment of COVID-19 is discouraged. 

Chlorpromazine is an anti-psychotic drug that, in addition to being an antagonist at dopamine and histamine receptors, also targets the cellular process of endocytosis. It is a phenothiazine derivative that like all phenothiazines, inhibits clathrin-mediated endocytosis via suppression of dynamin GTPase activity [54,55]. This property has been exploited successfully to inhibit viral (particularly CoVs) replication in vitro [56,57,58]. Based on these data, a phase III clinical trial “reCoVery” is being planned by a French group to test the efficacy of chlorpromazine in the treatment of COVID-19 [59].

Cepharanthine is a plant-derived alkaloid with strong anti-inflammatory effects and is approved in Japan for the treatment of several acute and chronic inflammation-related disease such as leukopenia, snakebite, xerostomia, and alopecia [60]. Its mechanism of action is multifactorial, and it has recently been reported that cepharanthine significantly increases endolysosomal pH in endothelial cells [61]. Exploiting this property, Fan et. al. [62] have reported that cepharanthine completely inhibits viral replication of the human SARS-CoV-2-related pangolin-CoV and is among the 24 drugs showing inhibitory effects on SARS-CoV replication in a cell culture model in vitro [63]. Therefore, it is proposed that cepharanthine may also inhibit human SARS-CoV-2 replication and could represent an optimal candidate for repurposing to combat COVID-19.

Altogether, targeting endocytosis is, at least in in vitro studies, an effective strategy, to prevent CoV infection. However, due to side effects, e.g., by treatment with hydroxychloroquine, their clinical application is still limited and further search for new targets is necessary.

## 4. Role of sAC in Lysosomal Function

### 4.1. Central Role of V-ATPase in Endocytosis

Many endosomal hydrolases function optimally at acidic pH. During endosomal maturation from early to late endosomes followed by fusion with lysosomes, endosomal pH is gradually lowered. The acidic pH of the endosomes and lysosome is maintained mainly through the activity of the vacuolar ATPase (V-ATPase) [64]. V-ATPases are large, multi-subunit complexes composed of an ATP-hydrolyzing peripheral domain (V_1_) and a membrane integral domain (V_0_) that translocates protons through the membrane. The activity of V-ATPase is controlled by several mechanisms. Regulated assembly of the V_1_ and V_0_ domains in response to a variety of cues such as nutrient availability, growth factor stimulation, and cellular differentiation rapidly modulates V-ATPase activity in mammalian cells [65]. Of note, infection of epithelial cells with influenza virus promotes V-ATPase assembly, which in turn increases the acidification of intracellular compartments, presumably as a means to facilitate cytoplasmic entry of the viral nucleic acid via low pH-mediated fusion of the viral coat with the endosomal membrane following endocytic uptake of the virus [66]. Likewise, overexpression of SARS-CoV protein 3CL^pro^ in the human monocytic cell line HL-CZ resulted in lysosomal acidification [67], suggesting an activation of host cell V-ATPase.

Aside from assembly, several mammalian cell types display regulation of V-ATPase activity via modulation of V-ATPase trafficking to the cell surface or to intracellular vesicles [68]. Among various signaling cascades able to regulate V-ATPase translocation to membranes, the role of the ubiquitous second messenger cyclic AMP (cAMP) has been addressed. Indeed, elevated cellular cAMP leads to translocation of V-ATPases to the apical surface of renal cells [69] via a protein kinase A (PKA)-dependent phosphorylation of the A subunit in the cytoplasmic V_1_ subunit of the V-ATPase [70]. 

### 4.2. Regulation of V-ATPase Trafficking by a sAC-Dependent cAMP Pool 

In mammalian cells, two distinct classes of adenylyl cyclase generate cAMP: G protein–regulated transmembrane adenylyl cyclases (tmACs) and biochemically distinct soluble adenylyl cyclase (sAC). sAC is constitutively active and is localized in the cytosol as well as within several cell organelles, including mitochondria and the nucleus; it can be synergistically activated by calcium and bicarbonate ions [71]. The distinct spatial distribution of sAC within the cell as well as restricted cAMP diffusion due to the activity of phosphodiesterases results in the formation of intracellular cAMP compartments. This compartmentalization is responsible for the specificity and selectivity of cAMP signaling. In contrast, cAMP produced by tmAC under physiological conditions is mainly localized close to the plasma membrane. Therefore, cytosolic localization of sAC provides a cAMP pool in proximity to the endocytic machinery and makes sAC a candidate for being involved in the regulation of endocytosis. Indeed, sAC is found inside the cell in a complex with V-ATPase [72]. Furthermore, a seminal study by Rahman et al. [73] revealed a key role of sAC in promoting translocation of V-ATPase to lysosomes. In mouse embryonic fibroblasts, the authors found that sAC promotes co-localization of V-ATPase with lysosomes, lysosomal acidification, and cathepsin-dependent lysosomal degradation. The study also revealed a potential role of PKA, although the role of other direct cAMP targets such as EPAC was not investigated. 

**Figure 1:**
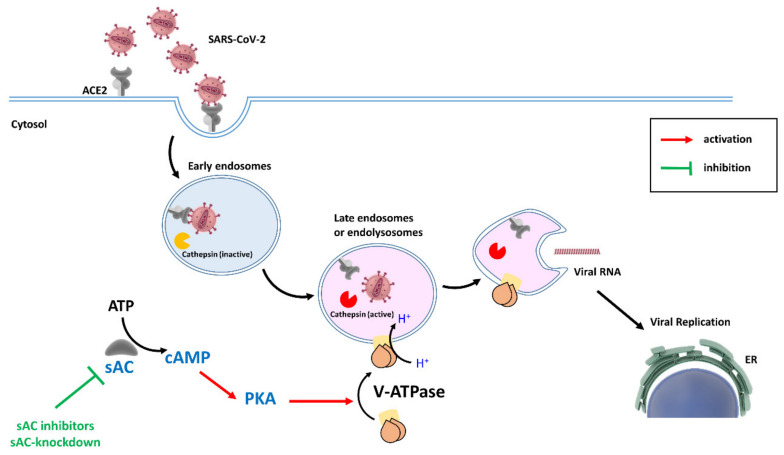
Hypothetical mechanism of sAC-dependent involvement in the entry of SARS-CoV-2 in host cells. The virus entry can be mediated by the endocytic pathway. Endosomal acidification resulting from the V-ATPase activity or fusion with lysosomes, and activation of cathepsin are key requirements for cleavage of the viral S protein and release of viral RNA into the cytosol. Since the sAC-generated cAMP pool may promote translocation of V-ATPase to endosomes and lysosomes, mediated by protein kinase A (PKA), sAC inhibition or knockdown may impair endocytic virus entry and, in turn, its replication. ACE2: angiotensin-converting enzyme 2. ER: endoplasmic reticulum.

In agreement with this study, we have recently revealed that sAC knockdown leads to a significant down-regulation of mitochondrial clearance in cardiac and endothelial cells [74]. These effects were associated with reduced lysosomal acidification and autophagosome accumulation (unpublished data), indicating disturbed autophagy flow due to lysosomal dysfunction. Altogether, the data suggest that sAC promotes lysosomal acidification in various cell types via V-ATPase co-localization with lysosomes. Since V-ATPase acidifies both endosomes and lysosomes [75], it is tempting to speculate that suppression of sAC expression or activity will interfere with the endosomal pathway of SARS-CoV-2 entry into the target cell. Figure 1 graphically presents the hypothesis of the present study.

### 4.3. cAMP signaling and CoVs 

Little is known about the role of cAMP signaling in modulating CoV infection and/or replication, although a few studies have provided some evidence in this regard [76,77]. In a study by Yamaya et al. [76], treatment of human nasal and tracheal epithelial cells with the β2 adrenergic receptor agonist formoterol suppressed HCoV-229E replication. Likewise, direct treatment with cAMP reduced viral replication and CD13 (aminopeptidase N) expression, a primary receptor for HCoV-229E. The authors suggest that formoterol may decrease HCoV-229E replication partly by modulating receptor (CD13) expression via cAMP production. In an investigation by Tao et al. [78] treatment of Calu-3 and Vero-E6 cells with a selective pharmacological inhibitor of EPAC (a direct target of cAMP) suppressed MERS-CoV replication; however, no mechanistic analyses were performed. Summarizing the data from these reports, it is evident that cAMP signaling may affect CoV infection in different ways. Whether cAMP signaling may affect the endocytic entry of CoVs, and particularly SARS-CoV-2, remains unknown. Considering the positive effect of sAC on V-ATPase activity and lysosomal acidification, one can speculate that inhibition of sAC may interfere with endocytosis and attenuate virus entry.

## 5. Role of sAC in Autophagy

Since V-ATPase leads to acidification of both endosomes and lysosomes, suppression of sAC, and, in turn, V-ATPase activity, may impair autophagy due to lysosomal dysfunction. Our recent studies support this idea by demonstrating that sAC inhibition or knockdown disturbs mitophagy in endothelial and cardiac cells [74], reduces lysosomal acidification, and leads to autophagosome accumulation (unpublished data). Furthermore, several studies have demonstrated the positive effects of cAMP elevation on the autophagy machinery [77,79,80]. 

The role of autophagy in viral infection by different CoVs, including SARS-CoV-2, has been investigated in several studies. In particular, the deletion of the essential autophagy genes ATG5 or ATG7 did not affect the replication of the murine hepatitis virus (MHV) [81,82] or SARS-CoV [83]. In contrast, MHV replication was impaired in ATG5-knockout embryonic stem cells [84]. Surprisingly, some studies showed the inhibitory effect of CoV on autophagy. For instance, a study using SARS-CoV and MERS-CoV in HEK293T, HeLa, and MCF-7 cells revealed that overexpression of membrane-associated papain-like protease PLP2 (PLP2-TM) of SARS-CoV and MERS-CoV led to a blockade of autophagosome and lysosome fusion and suppression of the autophagic flux [85]. Consistent with this, a recent report found that MERS-CoV blocks the fusion of autophagosomes and lysosomes, and induction of autophagy reduces the replication of MERS-CoV [86]. A very recent study from the same group, appeared on bioRxiv (not peer reviewed), showed that SARS-CoV-2 infection limits autophagy by interfering with multiple metabolic pathways, and that autophagy induction reduces SARS-CoV-2 propagation in vitro [87]. 

Taken together, the interplay between CoVs and autophagy is very complex and needs to be better investigated. Interestingly, a comprehensive review by Bello-Perez et al. [88] analyzing the effects of autophagy modulators on the replication levels of CoV in cell cultures revealed that the majority of the drugs tested, independent of their being autophagy inducers or inhibitors, show antiviral activity. These data suggest the importance of the autophagy balance for viral replication. Disturbance of the balance, i.e., promotion or inhibition of autophagy, interferes with viral replication. Since sAC inhibition may disturb the autophagy flow, one would expect an antiviral effect.

## 6. Role of sAC in Apoptosis

Aside from lysosomotropic effects, sAC inhibition may affect the SARS-CoV infection via modulation of host cell apoptosis. There are two main cellular mechanisms of apoptosis, the extrinsic (death receptor-dependent) and intrinsic (mitochondria-dependent) pathways. SARS-CoV infection leads to caspase-dependent apoptosis that can be prevented by overexpression of Bcl2 or by treatment with caspase inhibitor [89,90]. Similar results have been reported for SARS-CoV-2. In particular, cytological analysis of nasopharynx from COVID-19 patients showed markedly increased apoptosis and expression of active caspase 3 in glandular cells infected with SARS-CoV-2 [91], and SARS-CoV-2 infection in laboratory hamsters induced apoptosis in olfactory neurons [92]. Apoptotic mechanisms during SARS-CoV infection are likely to be manipulated by viral proteins. For instance, SARS-CoV proteins N, S, E, M, or 7a have been shown to induce apoptosis in the host cells [93,94,95,96,97]. Additionally, the SARS-CoV-encoded accessory protein ORF3a can induce apoptosis in different cell lines [8]. Of note, SARS-CoV proteins induce apoptosis via death receptor- or mitochondria-dependent pathways [8,93,96,97]. 

Aside from direct virus-induced apoptosis, excessive release of cytokines observed in COVID-19 patients, e.g., IL-1β, IL-6, and IL-10 [98], may lead to apoptosis in several cell types [10,99,100]. Interestingly, a negative correlation was found between cytokine concentration in the blood of COVID-19 patients and T cell numbers, which may be reduced due to cell death [101]. In another recent study, Chen et al. [102] explained this phenomenon by showing that the exposure to viral spike protein can trigger IL-6 gene transcription in macrophages in vitro. The authors suggest that macrophage-derived IL-6 triggers lymphocyte apoptosis and necrosis via enhancing Fas expression. Emphasizing the role of apoptosis in COVID-19 patients, a recent study revealed that SARS-CoV-2-induced severe lung damage occurs through cell pyroptosis and apoptosis [10]. In agreement, post-mortem analyses revealed apoptosis in endothelial and mononuclear cells in the lungs and small intestine in COVID-19 patients [16]. Thus, CoV infections, including that caused by novel SARS-CoV-2, lead to apoptosis of several cell types. As a result of cell death, reduced viral clearance by lymphocytes and epithelial/endothelial dysfunction accelerate the development of the disease. Of note, cytokines such as TNF-α and IFN-γ are known to down-regulate Bcl-2 expression, leading to release of mitochondrial cytochrome c and induction of the mitochondrial pathway of apoptosis [103,104,105].

Our previous studies emphasized the role of sAC in apoptotic cell death, particularly in the mitochondrial pathway, in various cell types induced by oxidative stress, ischemia, or oxysterols [106]. The main sAC-controlled apoptotic pathway consists of PKA-dependent phosphorylation/activation of the pro-apoptotic Bax protein followed by its translocation to mitochondria, the release of cytochrome c, and cleavage of caspases-9 and -3. In addition, sAC is also involved in activation of another pro-apoptotic protein, Bad, a component of the mitochondrial pathway of apoptosis [107]. Although there are no data showing activation of mitochondria-dependent apoptosis by SARS-CoV-2, several reports concerning other CoVs argue for the role of mitochondrial apoptosis in virus-induced cell death [93,96]. Noteworthy, several studies with different CoVs showed that viral infection or viral protein expression triggers Bax translocation to the mitochondria, the release of mitochondrial cytochrome c into the cytosol, and apoptosis [108,109]. Thus, the data indicate that suppression of sAC may improve cell survival and support immune clearance of the virus.

## 7. Conclusions and Perspectives

The COVID-19 pandemic has elicited an urgent search for novel antiviral drugs to treat SARS-CoV-2 infection. Targeting the sAC-dependent cAMP pool may be a promising approach primarily because of its effects on the endocytic pathway of viral entry via promoting V-ATPase-dependent acidification of endosomes and lysosomes. Previous in vitro studies showed that the impairment of the endo-lysosomal acidification with bafilomycin A1 or chloroquine suppresses viral infection [42,46]. However, recent clinical studies [52,53,110] showed the severe side effects of chloroquine or hydroxychloroquine that may compromise survival of COVID-19 patients. 

The selective inhibition of sAC activity may provide an alternative strategy to combat viral infection by targeting endocytosis. Aside from endocytosis, suppression of lysosomal acidification also interferes with autophagy, whose role in CoV-induced infection is still controversial. In contrast, suppression of apoptosis via sAC inhibition may be beneficial in the treatment of COVID-19 patients, as it may improve virus clearance by lymphocytes, due to the better survival of lymphocytes, and endothelial/epithelial survival and function in the lungs and other organs. 

sAC is widely expressed and is involved in several cellular pathways and physiological functions [71]. Thus, local or systemic sAC inhibition may potentially lead to several unwanted side effects. Nevertheless, the main phenotype in the two molecularly distinct sAC knockout mouse strains is male-specific sterility [111,112]. Interestingly, two male patients were discovered recently to be homozygous for a rare mutation in the coding region of ADCY10 gene (sAC) that leads to premature termination and interruption of the catalytic domains of sAC [113]. Aside from infertility, the only reported health issue in these men is an increased incidence of kidney stones. Some other phenotypes described for the sAC knockout mice [114] are not expected to be detrimental when transiently induced (i.e., increased risk of kidney stones, increased intraocular pressure, decreased leukocyte migration). Thus, the effects of temporary sAC inhibition may be primarily restricted to male infertility, and it appears that changes in somatic functions of sAC-generated cAMP are likely to be tolerated.

Efforts have been made to develop selective sAC inhibitors [114]. A recently developed, non-toxic sAC-selective inhibitor LRE1 [115] is precluded from further development due to its numerous flaws, including poor bioavailability (<5%), high intrinsic clearance, and high rate of metabolism [116]. A more recent effort, undertaken to develop new male non-hormonal, orally available contraceptives as a collaboration between academia and a public-private drug discovery institute [116], provides hope for the development a new sAC-selective inhibitor applicable in the clinical setting.
